# In-utero exposure to opioid increases the risk of congenital heart defects

**DOI:** 10.17179/excli2020-1051

**Published:** 2020-03-02

**Authors:** Kamleshun Ramphul, Stephanie G. Mejias, Jyotsnav Joynauth

**Affiliations:** 1 Department of Pediatrics, Shanghai Xin Hua Hospital, Shanghai Jiao Tong University School of Medicine; 2 University Iberoamericana UNIBE School of Medicine, Dominican Republic; 3 Children's Hospital Zhejiang University School of Medicine, Hangzhou, Zhejiang, China

## ⁯

***Dear Editor, ***

The global incidence and burden of Neonatal Abstinence Syndrome (NAS) are constantly increasing (Ramphul et al., 2020[4]) due to the rise in opioid use in different societies. There are various studies linking the use of opioid during pregnancy with multiple growth and developmental defects in the fetus and newborn. We performed a retrospective study to analyze the risks of several congenital heart defects with newborns that were exposed to opioid during fetal life.

The Kids' Inpatient Database (KID) is the largest hospital-based pediatric database in the United States. It is compiled by the Healthcare Cost and Utilization Project (HCUP) and sponsored by the Agency for Healthcare Research and Quality and their partners (https://www.hcup-us.ahrq.gov) (HCUP, 2019[3]). The 2012 KID was used in our study. All neonates with a diagnosis of neonatal abstinence syndrome were identified using the International Classification of Diseases, Ninth Revision, Clinical Modification (ICD-9-CM) code “779.5”. Since Down syndrome is a major risk factor for congenital heart diseases (Ramphul et al., 2019[5]), we excluded all cases of Down syndrome from this study.

We performed Chi-square tests and logistic regressions on SPSS 20 to calculate the risks of ventricular septal defects (VSD), tetralogy of Fallot (TOF), atrial septal defect (Ostium Secundum defect) (ASD), and coarctation of aorta in neonates presenting with NAS. 

A total of 22145 (weighted) neonatal abstinence syndrome patients were considered suitable for our study. 305 (1.6 %, p<0.05, OR: 2.897, 95 % Confidence Interval 2.585-3.247 ) cases of VSD, 26 cases (0.1 %, p<0.05, OR: 2.376 , 95 % Confidence Interval 1.615-3.495) of tetralogy of Fallot, 1248 (5.6 %, p<0.05, OR: 3.870, 95 % Confidence Interval 3.653-4.100) cases of ASD, and 47 (0.2 %, p<0.05, OR: 2.700, 95 % Confidence Interval 2.023-3.604) cases of coarctation of aorta were identified (Table 1(Tab. 1)).

The pathophysiology behind the teratogenic effects of opioid on cardiovascular development is still not fully understood. Opioid use during fetal life has shown to impact the migration and development of various systems such as neurons in rat studies and DNA synthesis in fetal cardiac tissues (Walhovd et al., 2009[6]; Broussard et al., 2011[2]). While the HCUP database has some limitations, our results strongly support previous studies highlighting opioid use as a possible risk factor for congenital cardiovascular defects (Brennan and Rayburn, 2012[1]). 

## Conflict of interest

The authors declare no conflict of interest.

## Figures and Tables

**Table 1:**
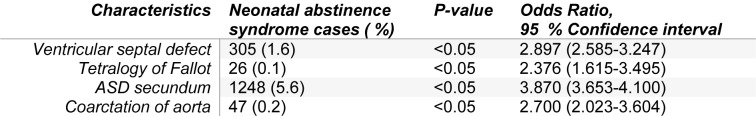
Characteristics of multiple cardiac defects in children with Neonatal abstinence syndrome

## References

[1] Brennan MC, Rayburn WF (2012). Counseling about risks of congenital anomalies from prescription opioids. Birth Defects Res A Clin Mol Teratol.

[2] Broussard CS, Rasmussen SA, Reefhuis J, Friedman JM, Jann MW, Riehle-Colarusso T (2011). Maternal treatment with opioid analgesics and risk for birth defects. Am J Obstet Gynecol.

[3] HCUP (2012). Kids’ Inpatient Database (KID). Healthcare Cost and Utilization Project (HCUP). Agency for Healthcare Research and Quality. www.hcup-us.ahrq.gov/kidoverview.jsp.

[4] Ramphul K, Mejias SG, Joynauth J (2020). An update on the burden of neonatal abstinence syndrome in the United States. Hosp Pediatr.

[5] Ramphul K, Mejias SG, Joynauth J (2019). Down syndrome predisposes to congenital cardiac malformations. EXCLI J.

[6] Walhovd KB, Moe V, Slinning K, Siqveland T, Fjell AM, Bjørnebekk A (2009). Effects of prenatal opiate exposure on brain development - a call for attention. Nat Rev Neurosci.

